# Researches into the Physical History of Mankind

**Published:** 1837-04

**Authors:** 


### Art. IV.

*Researches into the Physical History of Mankind.* By James Cowles Prichard, m.d. f.r.s. &c. &c. *Third Edition.* Vol. I.—*London,* 1836, 8vo. pp. 376.

It is an interesting fact in the history of medical literature, that several of the most valuable works, and those which have extended far and wide the fame of the authors, have originated in an inaugural thesis. An accidental direction having been given to the student's attention in early life, the germ of the future work has often existed in the academical dissertation, and each new edition has manifested the greater extent of enquiry, the increased learning, the wider observation, and the more matured judgment of the writer. Such, we believe, was the origin, and such has been the progress, of the work before us; a work already celebrated, and of a scope so extensive, and so full of minute information, as almost to deter a mere medical critic from giving an account of it.

In the ten years which have elapsed since the appearance of the *second* edition of this work, numerous contributions have been made to the Physical History of Mankind; but we have seen no comprehensive work which has recalled our attention to the whole of this vast subject, until the appearance on our table of this, the first volume of Dr. Prichard's third edition. The progress of the general science of natural history has, however, been very great within this short period, not only with the public, but among medical men. The ungenerous notion, formerly so successfully disseminated, that no medical man should attempt to be a man of science, exists no longer. A medical man cannot preserve his intellectual rank in society without some scientific acquirement, and subjects which were once almost confined to the closets of philosophers are now discussed at dinner tables, declaimed upon in drawing-rooms, and settled at conversazioni. So far from considering these circumstances as indicative of a general superficiality of acquirement, we regard them as proofs of some acquirement where formerly there existed none; and as belonging to that general uplifting of all classes in the scale of knowledge which, to say the least of it, may be contemplated without disdain. One result of this advancement of science will be, we doubt not, that for every reader of the previous editions of the work before us there will be ten readers of the present one.

The physical history of man was " first explored," in the words of Dr. Prichard's dedication, by " the venerable and universally celebrated Blumenbach." Before his time, no work of any consequence had appeared on the comparative anatomy of the human races, and he was the first to make a collection of human skulls sufficiently extensive to illustrate important diversities of structure. Dr. Prichard himself was the first to institute an express enquiry into the comparative physiology and psychology of different races of men, his inaugural essay being published in 1808, and the first edition of his larger work in 1813. Throughout these successive publications, Dr. Prichard has maintained the unity of the species in all the races of mankind; a doctrine supported by Mr. Lawrence in his celebrated lectures, but opposed by Rudolphi, Virey, Desmoulins, Bory de Vincent, and other writers, who contend for an original diversity of races; the latter opinion being countenanced by Cuvier, and, Dr. Prichard thinks, by De Humboldt, De Spix, Martius, and several distinguished French navigators and naturalists. Notwithstanding this formidable array of names, Dr. Prichard's faith in the opinion of the unity of the species remains unshaken, and his work may be considered in the light of an immense collection of evidence in favour of it. The volume before us is divided into two books, whereof the first is devoted to the consideration of the *Origin and Dispersion of Organized Beings*, and the second to an *Analogical* investigation of the *Identity or Diversity of Species.*

The difficulties attendant on the whole of this enquiry are great, and yet not obvious, except to those who have made some advance in it. Ordinary observers would be inclined to do what philosophers have often been content to do, namely, seeing the evident and striking diversities of mankind, to take it for granted that the origin of the different races must have been as diverse as are their external characters, their complexion, form, and habits of life. A person imbued with no previous theories, but to whom the whole spectacle of human diversities was at once exhibited, would perceive the difficulty of referring such remarkable varieties to one stock or commencement.

"If such a person, for example," says Dr. Prichard, "after surveying some brilliant ceremony or court-pageant, in one of the splendid cities of Europe, were suddenly carried into a hamlet in Negroland, at the hour when the sable tribes recreate themselves with dancing and barbarous music, or if he were transplanted to the saline plains over which bald and tawny Mongolians roam, differing but little in hue from the yellow soil of their steppes, brightened by the saffron flowers of the iris and tulip,—if he were placed near the solitary dens of the Bushmen, where the lean and hungry savage crouches in silence, like a beast of prey, watching with fixed eyes the birds which enter his pitfall, or the insects and reptiles which chance may bring within his grasp;—if he were carried into the midst of an Australian forest, where the squalid companions of kangaroos may be seen crawling in procession, in imitation of quadrupeds;—would the spectator of such phenomena imagine the different groups which he had surveyed to be the offspring of one family? and, if he were led to adopt that opinion, how would he attempt to account for the striking diversities in their aspect and manner of existence?" (P. 1.)

To solve the great question which would suggest itself, several subordinate questions must be entertained, relating to physiology, to the nature of intellectual and moral diversities, and to the origin and formation of languages; questions for the elucidation of which it is not necessary for us to remind the reader that Dr. Prichard is eminently qualified by the rare combination of the learning of a scholar with the range of observation and practical knowledge of a philosopher. This kind of enquiry is followed, although not in the volume now before us, by an historical examination of the *changes* which have actually arisen in the physical characters of nations or human races;—a portion of the enquiry which Dr. Prichard terms the ethnographical. The bearing of both these modes of enquiry on the question of the identity or separateness of the human species will readily be understood. He enters upon the whole task with the candour of a well-prepared augmentator, placing before us the difficulties attendant on his own theory, and their apparent removal by the admission of the supposition of a variety of origins; by which we should seem at once able to explain diversities of colour, the causes of which are obscure, and also to reconcile the fact of many distinct forms of speech prevailing in different parts of the world with that of the similarity traceable between the existing Egyptian language and that which was known in the days of Joseph or of Abraham. The striking differences in the moral and intellectual qualities of different races, the discovery of inhabitants in lands before unknown, and of some of them the barbarous or primitive customs, and the remains, even among such people, of a race anterior to them, and extinct, and of whom no other memorials exist, would all seem to be accounted for by the supposition "that each distant country was originally provided by nature with a peculiar stock of home-born inhabitants."

Against such a supposition, Dr. Prichard observes, might be set the authority of the Sacred Writings, of which, however, he considers that he cannot with propriety avail himself in such an enquiry as he proposes. But he still considers the supposition as untenable, and proposes to combat it on other grounds.

Proceeding to the first part of his enquiry, or that which relates to the origin and dispersion of organized beings, the question is discussed, "Whether, throughout the organized world, including both its great departments, it has been the method of nature (if that expression may be used,) to produce at first only one family in each particular species, or to call beings of the same specific structure into existence simultaneously from different beginnings, and to diffuse them over the world from many distinct centres or original points." Some of the most probable of the different hypotheses which have been maintained on this subject are examined; that of Linnæus, that all animals and all plants originated in one common birthplace in some fertile region of the earth, the only tract as yet laid bare by the subsidence of the primeval ocean, being dismissed as "more allied to poetry or fiction than to a serious investigation of the phenomena of nature." Two other hypotheses, concerning which the botanists of the present day are divided, are carefully considered; the first being that every species or distinct tribe of vegetables had a distinct centre or birthplace, but that their first centres or birthplaces were in different regions of the earth; and the second being, that the vegetable tribes are universally spread over the earth, and brought into existence wherever circumstances favour them. The latter conjecture is considered by Dr. Prichard as not being more tenable than that of Linnæus. Districts of the globe having a parallel temperature and elevation, a similar soil, and equal humidity, are found to produce species of plants which are *not* identical; and plants become naturalized in countries to which they have been conveyed by human agency, but where they were never produced before. The probable conclusion at which Dr. Prichard arrives, after an enquiry very full of interest, but which we cannot attempt to abridge in these pages, is, "that each tribe of plants, and especially of the more perfect plants, had on the earth one original habitation, from which it has been dispersed according to the capabilities afforded by its structure, and the aid of external agencies." (P. 53.)

Those who have not attended to this subject are little aware of the provision made for the extensive dispersion of seeds. They are fully considered by Dr. Prichard. Among them human agency is very important. Wherever human beings exist, some vegetable clings to life or flourishes in the sun, furnishing food, or clothing, or the materials of dwellings, or grateful shade. In the intentional transportation of the most useful kinds, others are accidentally conveyed, and often to distant parts of the earth. When rice or other grain is imported for food, the knap-weed, the Erigeron Canadense, and other plants, intrude themselves unsought; and sometimes the intruders, as is the case of the thorn-apple and the hemp, prove as useful as the seeds they accompany. The wild plants of Africa come to Europe with the wheat of Barbary, and take root in the south; and near the gate of Montpelier, where foreign wools are dried, foreign plants, which have adhered to the fleeces, find nourishment in the soil, and spread out their leaves in a strange land. The seeds of plants adopted from the wilds of North America, and cherished in the gardens of France, have been blown by the winds of heaven to every temperate region of Europe; and the plants of Europe fertilize and adorn the Cape of Good Hope. This kind of dispersion has occasionally led to curious observations. In the neighbourhood of Warminster, we read in the Philosophical Transactions, the good people were, more than a hundred years ago, surprised by what they deemed to be a shower of wheat; which, however, proved to be ivy-berries. One of the versions of the time was, that they were "found in the hail, as seeds in comfits."

The agency of animals is also subservient to the diffusion of vegetable productions. Birds not only swallow and eject seeds without impairing their vitality, but some seeds are rendered even more fit for vegetation by this process. Such is stated by Mr. Lyell to be the case as respects the seeds of the white-thorn; the growth of the quickset being hastened if the seeds have previously traversed the digestive canal of turkeys.

But the atmosphere and water are the great means of the diffusion of seeds. It is a subject of popular observation that new plants will make their appearance when a portion of ground has been laid bare. These are sometimes the product of seeds which seem to have been long buried in the ground, but often, Dr. Prichard maintains, merely produced by the deposition of seeds by the atmosphere in soils rendered congenial to them by the new process. Wherever wood-ashes are strewn, or weeds are burnt, or soap-lees spread on the ground, clover will make its appearance, and white clover follows the drying, and liming, and breaking up of the Scotch hill-pastures; and in all these cases, the vegetable or mineral alkali is supposed to have made the earth a congenial receptacle for seeds which floated in the air. So also, the growth of some plants is known to prepare soils for the reception of other species. When the great pine-forests of America are destroyed by fire, they are succeeded by forests of oak. It must, however, be confessed, that in the instances in which seeds could not have been recently introduced, Dr. Prichard is compelled to remain satisfied with the mere conjecture of their having long remained buried in the earth; and to this cause, as well as to that of the same subterraneous cryptogamous plants being found in the mines of New Spain as grow in deep excavations of the earth in Europe, although Dr. Prichard's views do not want support in the evident traces, of other kinds, of ancient inundations and changes, which must have affected the vegetable as well as the mineral world,—the opinion of Rudolphi would probably afford a more satisfactory solution to many readers; namely, that the seeds of plants are universally diffused, and grow wherever certain conditions exist essential to their growth. But that the light seeds of numerous plants are carried far by the winds is well known; and the structure of the seeds seems evidently adapted to such flights in many instances. M. de Candolle found two lichens of Jamaica on some trees on the south-west coast of Britanny, the seeds of which he supposed to have been brought across the Atlantic during the long prevalence of south-westerly winds.

Many seeds float upon the waters; and those of the sea-coast especially are transported to the coasts of other countries. The seeds of tropical plants are thus collected in the Hebrides. The Alpine streams scatter on the banks of rivers in the plain the seeds of mountain-plants. The rivers of Germany carry seeds from the interior to the Baltic; and on the western shores of the Atlantic are found those of the interior of America.

Having come, then, to the conclusion that each tribe of plants, or at least of the more perfect tribes, had its own original place of habitation in the earth, from which it has been more or less dispersed, Dr. Prichard proceeds to extend a similar mode of enquiry to the lower animals, before entering upon it with respect to mankind. On the threshold of this enquiry, he meets with the fact respecting animals, so analogous with what he had previously mentioned respecting plants, that whilst those of greatest bulk and most complex organization seem limited to certain portions of the earth, those of the smallest size and simplest structure are almost everywhere dispersed. Dr. Prichard does not conceal the difficulty of applying here the explanation offered when speaking of the universal dispersion of the lower orders of plants, nor the ridicule thrown by Rudolphi on the hypothesis of the ova of such animals being diffused through the atmosphere. He refrains, however, from entering fully on the difficult question of equivocal or spontaneous generation, which, he observes, is at least not required with regard to the higher order of animals; whilst, with regard to the lowest, the experiments of Spallanzani and Sennebier prove "that, by precluding the access of minute seeds and ova floating in the atmosphere, the production of minute animal and vegetable life is prevented, in a very great measure at least, from taking place." (P. 55.)

So many insects depend for existence on particular plants, that their dispersion may reasonably be supposed to follow the same order, and often to be produced by similar causes. No insects are universally diffused. But the argument rests more securely on the facts known respecting birds: those of weakest flight are found confined to the narrowest limits, as the parrot tribe, the humming-birds, and the common grouse of England; a fact only explicable by the theory which supposes their original habitation to have been distinct. As regards cetaceous animals and fishes, the limits within which particular species are confined appear to be still more generally marked, and with a smaller number of exceptions. The mammiferous animals and quadrupeds seem even more palpably to be limited to certain provinces, to which they are partly confined by the difficulties of egress, and partly by climate. The region round the North Pole is one of these provinces containing "tribes of quadrupeds common to all the northern regions of the world." Thus also certain quadrupeds exist in the temperate zone, and range from the western to the eastern extremities of the old continent, whilst the quadrupeds of the same zone in the New World are entirely different. The equatorial zone, divided into three great tracts by wide seas, impassable to quadrupeds, contain three distinct assemblages of animals, the American, the African, and the Indian.

"Fourthly. The great and numerous islands of the Indian Archipelago, separate, and perhaps anciently torn from the continent of Asia, form, with reference to their geographical position, a distinct region of the earth, which is similar in climate and in its vegetable productions to the hottest parts of Africa. Here we shall expect to discover mammifers and reptiles of peculiar character.

"Fifthly. Beyond the Indian Archipelago, we find a remarkable country, very fertile in vegetable productions. Papua includes New Guinea, New Britain, and New Ireland. The lofty mountain ranges which support it spread themselves out in several great arms, and run southwards into the Pacific, forming straits and groups of islands, everywhere attaining a considerable elevation. The Archipelago of Solomon, the Arsacidæ, Louisiade, Santa Cruz, Tierra del Espiritu Santo, the New Hebrides, New Caledonia, and perhaps the two long islands of New Zealand, appear to be branches of the same central region.

"The more remote groups of islands in the great Southern Ocean may be reckoned as parts of the same zoological province. The whole of this region is placed under circumstances likely to call forth the most abundant productions of organized nature. In fact, its vegetation is luxuriant; but, as we might expect to find, in conformity with the preceding observations, the animal creation is as remarkably deficient in its principal tribes.

"Sixthly. Beyond the Indian seas, but separated from New Guinea only by straits, we find an extensive continent, differing from the rest of the world in all its physical peculiarities. Terra Australis, or Austral Asia, according to the best informed observers, is peculiar and striking in its geological aspect. It is equally remarkable for the singularity of its vegetation. In no part of the world has the animal creation so distinct and peculiar a character.

"Seventhly. The southern extremities of America and of Africa contain countries situated under a similar climate. The temperate parts of these continents, as well as that of Terra Australis, are thus so many insulated regions. In all we may expect to find peculiar tribes." (P. 70.)

Dr. Prichard devotes a section to the consideration of the peculiar characters of each of these great zoological provinces, in which are enumerated many facts very interesting to the naturalist, and all bearing on the great questions of the dispersion of animals from one common centre, their universal diffusion, or their location in particular latitudes, and dispersion within such zone as widely as physical obstacles would allow. The first of these is dismissed, as involving difficulties which amount to physical impossibilities, and as "contradicted by the uniform tenour of facts, both in botany and zoology." The second, also, is put aside as inadmissible. With respect to the third, Dr. Prichard thus expresses himself, and with these words concludes the preliminary portion of his enquiry.

"The inference to be collected from the facts at present known seems to be as follows:—the various tribes of organized beings were originally placed by the Creator in certain regions, for which they are by their nature peculiarly adapted. Each species had only one beginning in a single stock; probably a single pair, as Linnæus supposed, was first called into being in some particular spot, and the progeny left to disperse themselves to as great a distance from the original centre of their existence as the locomotive powers bestowed on them, or their capability of bearing changes of climate, and other physical agencies, may have enabled them to wander.

"The bearing of this general conclusion on the enquiries hereafter to be pursued is sufficiently obvious. We have now to investigate the question, whether all the races of men are of one species in the zoological sense, or of several distinct species. If it should be found that there is only one human species in existence, the universal analogy of the organized world would lead us to the conclusion, that there is only one human race, or that all mankind are descended from one stock. It is the more improbable that a plurality of races exists in one species with reference to man than with regard to any inferior tribe, as the locomotive powers of mankind, aided by the resources of human sagacity, are greater than those of brute animals." (P. 96.)

The philosophical spirit in which Dr. Prichard entered upon the extensive enquiry which constitutes the subject of the work, is, the reader cannot but observe, signally attested by the circumstance that, up to this stage of it, all the analogies are against the view he ultimately takes of the origin and dispersion of mankind. Impressed with the facts resulting from an investigation of the origin and dispersion of plants, and still more of the lower animals, up to man himself, the imagination readily forms to itself imaginary zones or boundaries within which human beings, like the mammifers, may have been created, and to which their physical constitution is suited; their range being enlarged beyond that of the animals by superior endowments, and greater power, resulting from those endowments, of overcoming the physical obstacles which imprison the most powerful quadrupeds. The mere aspect of the different varieties of mankind comes so strongly in support of this imagination, as perhaps not wholly to leave the mind free from its influence after reviewing the largest collection of facts and the strongest arguments in disproof of it.

A very material element in this whole enquiry concerning man's descent from one or many stocks, is the determination of the singleness or multiplicity of the *species* of men as they now exist, however various the primary difference which so forcibly strikes the senses, and maintains so much power over the mind of observers. The difference of species observed as relates to the phocaceous animals found in the southern and northern hemispheres obviously favours the reception of the opinion that each species was created in the zone in which it is found. The division, therefore, of the human race into two or more species, or its union in one, everywhere to be recognized under adventitious disguises, is most important to the great question which Dr. Prichard has so elaborately elucidated. It is, therefore, most essential that the reader should understand the precise signification of the term species, and we give it in the author's own words:—

"The meaning attached to the term *species* in natural history is very definite and intelligible. It includes only the following conditions, namely, separate origin and distinctness of race, evinced by the constant transmission of some characteristic peculiarity of organization. A race of animals or of plants marked by any peculiar character which has always been constant and undeviating, constitutes a species; and two races are considered as specifically different, if they are distinguished from each other by some characteristic which the one cannot be supposed to have acquired, or the other to have lost through any known operation of physical causes; for we are, hence, led to conclude that the tribes thus distinguished have not descended from the same original stock." (P. 105.)

Dr. Prichard observes that the term *genus* was formerly used with the same signification, but that it became, as well as the English word *kind*, at length to be applied too comprehensively; the expressions ox-kind, dog-kind, cat-kind, &c., comprehends assortments of animals clearly distinguished from other groups, but yet not all of one species. No physical causes are known to us which are capable of producing the different breeds comprehended in the horse-kind or oxen-kind, or dog or cat-kind, although their general model has led many to entertain the opinion, founded merely on conjecture, that the different species each of these kinds comprehends were originally identical.

Within the limits of each species there exist *varieties*, produced by the action of external causes, and such varieties are congenital and hereditary.

"*Varieties* are distinguished from species by the circumstance that they are not original or primordial, but have arisen within the limits of a particular stock or race. *Permanent varieties* are those which, having once taken place, continue to be propagated in the breed in perpetuity. The fact of their origination must be known by observation or inference, since, the proof of this fact being defective, it is more philosophical to consider characters which are perpetually inherited as specific or original. The term permanent variety would otherwise express the meaning which properly belongs to species. The properties of species are two; viz. original difference of characters and the perpetuity of their transmission, of which only the latter can belong to permanent varieties." (P. 109.)

Before attempting to follow the author through his enquiry, it is quite essential that clear ideas should be imbibed concerning these distinctions. They are equally necessary to all who have opportunities of gathering evidence on the subject themselves; and we have dwelt more particularly on the preliminary portion of Dr. Prichard's work on this account; for, when we look at the mass of illustrative evidence which he lays before the reader, we see the impracticability of giving more than a brief and general view of it. The question being, whether all the visible diversities of mankind are referrible to varieties only, or arise from differences of species, Dr. Prichard enters first into an extensive physiological and anatomical investigation, and a review of phenomena relative to the origin of varieties in breeds, so as to determine, or attempt to determine, what is permanent and what subject to change in any species; and then upon a survey of the different races of men, in order to determine the changes that have actually arisen in the physical characters of nations or human races; a task of vast extent, and yet full of interest for students of almost every description.

In proceeding to it, Dr. Prichard alludes to the fact, a knowledge of which has arisen from his investigations, that the most permanent characters in the animal kingdom are functional rather than those of structure; and that the principal structural varieties which take place are in the external and less essential parts. Then, directing his attention to the laws of the animal economy, he observes that they are found remarkably uniform in animals of the same species, but vary greatly in animals which, although resembling each other, are yet specifically distinct.

"Thus the wolf and the dog, though proximate species, differ remarkably as to the period of utero-gestation. The she-wolf appears to carry her young ninety days, while the period of gestation in the bitch is well known to be sixty-two or sixty-three days; the former being longer by nearly one-third part than the latter. We are acquainted with no similar deviation in the animal economy within the limits of any one species, and it is very unlikely that such a fact will ever be observed." (P. 115.)

This observation is illustrated by the inference deducible from Dr. Prichard's enquiries concerning the longevity of man in different parts of the world, which appears in all countries to be tolerably uniform, allowance being made for obvious causes of decreased life; whereas, the term of life reached by the longest lived of the simiæ, and those which most nearly resemble man, (as the chimpanze or troglodyte,) is only about thirty years. An equally careful enquiry into the progress of physical development, the periodical phenomena of the constitution, and the state of the natural and vital functions in different races, shows that there are, in these respects, no such differences existing between the different varieties of mankind as to raise the question of diversity of species: the specific temperature, and the action of the heart and pulse, are the same, and the development of the body takes place in similar periods, with some modifications, obviously the effects of climate.

Notwithstanding the capability of procreation found to exist in several instances of hybrids, there is evidently existing a provision against such a result in general, so that the species of animals are kept distinct, and the order and also the variety of animals continues to be universally preserved. Dr. Prichard enquires into the nature of this provision, and considers that it consists of several different circumstances, as the mutual repugnance between animals of different species, and the consequent rarity of mule productions; the infrequency of the progeny being matured; and the general sterility of hybrids; and the difficulty, amounting in a few generations to an impossibility, of keeping up such a stock, even by a reunion with a pure breed. With these facts Dr. Prichard compares the well-known results of crossing or intermixing varieties or races belonging to the same species, and only differing in colour or form; the produce of such crossing being often superior to the parent animals; and he then proceeds to the question as regards the mixed races of the human kind, "whether they are, in the phenomena of their propagation, analogous to hybrid productions or to the blended offspring of tribes which are merely varieties of the same species," (p. 147;) and he adduces some convincing examples of the improvement of mixed races, or races arising from parentage of different colours or tribes, the progeny being found to be more vigorous, and to have a greater tendency to multiply. Dr. P. does not lay undue stress on this part of his argument, but considers it "manifestly favorable to the doctrine that the several tribes of man are but varieties of the same species." (P. 150.)

Another head of the investigation regards the proofs deducible from pathological considerations; proofs necessarily imperfect in the present limited state of knowledge respecting the diseases of animals. But, after comparing the different races of human beings as regards their proneness to be affected by contagious, epidemic, and endemic diseases, Dr. Prichard concludes that the great catalogue of diseases are common to the whole human family, although differing in different climates, and although local circumstances may engender peculiar predispositions to disease in races long exposed to them; so that "the pathological history of different races appears to illustrate and confirm the inference, already deduced from researches into their physiology, that a common nature belongs to all mankind." (P. 160.)

No part of the analogical investigation pursued by Dr. Prichard is likely to prove more generally interesting than that which touches the psychical characteristics of mankind. It abounds, indeed, with details of a description which no observer of mankind or friend of man's improvement can read altogether unmoved. To many it may be new to learn the virtues of the Bushmen or Hottentots, or the aspirations of the Esquimaux after immortality; and the analogy between the superstitions of the Negroes and some of the doctrines for which much blood has been shed in nations boasting of peculiar civilization, cannot but excite a lively surprise. Sacrifices, the worship of animals, offerings of cattle and of fruits, pilgrimages, oracles, auguries, holy aspersions and inunctions, and all the peculiarities of classical and of modern superstition, are found among the Negroes; and, in the doctrine of the metempsychosis, even the wretched slave enjoys a promise of a renewal of life, supported by which he voluntarily terminates his natural existence, in the hope of being again a happy infant, and of beholding his native land once more. The notices of the conversion of some of the natives of these various countries to Christianity, are deeply affecting; and to them, as indeed to every line of this section, we strongly recommend the reader's earnest attention. Medical students may not require to go over the proofs of the separateness of the human species from the apes, but all may read with improvement, not mental only, but of the feelings, the proofs, not less undeniable, that, however distinguished by colour or some modifications of external structure, whether basking in luxurious refinement or contending in a savage state for a precarious existence,—whether enjoying all the blessings of liberty, and civilization, and knowledge, or condemned to pine in slavery and the grossest ignorance,—the human being is still distinguished above all the animals by moral and mental endowments strictly identical; and, making a reasonable allowance for degrees of development, resulting from climate and from the different stages of civilization, possesses everywhere the same faculties of the mind,—is everywhere agitated by the same emotions, and from every corner of the earth directs the same hope towards a great First Cause, and towards a future existence;—everywhere, in short, affords proofs of similarity of species and a common origin.

The analogical investigation is extended from these topics to the visible or external diversities of the races of man, as variety of complexion, and to those of form and structure, and of diversities observable in the skeleton; and with these are compared the varieties observed in other parts of the creation, as of colour, texture of skin, &c.; and the laws of the transmission of varieties are examined.

Dr. Prichard adopts the division of mankind into three classes, distinguished by the colour of their eyes and hair; as the *Melanocomous*, or black or dark-haired; the *Xanthous*, having yellow, or red, or light-brown hair, and blue or light-coloured eyes; and the *Leucous*, or albinos. He considers the varieties of form and structure as dividing mankind into seven classes, separated by strongly marked lines; among which are peculiar forms of the skull. The first of these classes comprehends those nations which resemble the Europeans in the shape of the skull and in other physical characters; as many nations in Asia, and some in Africa. The second class contains those races which resemble in these respects the Kalmucks, Mongoles, and Chinese. These classes are denominated by Dr. Prichard as Iranian and Turanian nations; and he altogether avoids the terms Caucasian and Mongolian. The native American nations constitute the third class; and the Hottentot and Bushman race the fourth; the Negroes make the fifth class; the sixth comprises the Papuas, or woolly-haired nations of Polynesia; and the seventh consists of the Alfourou and Australian races.

We find it impossible to condense the numerous particulars relating to this part of Dr. Prichard's enquiry, or to quote any passages which could be satisfactorily presented to the reader disjoined from what goes before or follows them. Each of the classes is separately examined; a very instructive chapter is devoted to the national forms of skulls, and another to the form of the skeleton. The comparative stature of different races, and the laws which govern the transmission of varieties are also considered. All these portions of the work require to be carefully read, as upon their evidence the whole conclusion of the analogical enquiry rests.

The conclusion (with which the first volume terminates) is, that the various colours of the different races of mankind do not present obstacles to considering all the races as descended from one stock; the counterparts of them being found in most tribes of domestic animals. We might even expect much greater varieties in man than in animals, taking into consideration the greater variety of circumstances, physical and moral, to which he is exposed, in the greater range of regions over which he can wander, and wherein he can support existence. Nor do the remote correspondences maintained by some writers to exist between the skeleton of the negro and the orang impede this view of such variety; for, in the animal kingdom, analogous circumstances are observed, as in particular breeds of dogs, which resemble the wolf in structure, and in other instances. It may yet be asked by some of his readers, Dr. Prichard observes, if these phenomena of variety depend on similar causes in mankind and in the lower animals; and also whether they are more permanent in man than in the lower tribes. The answer to these questions is thus given:

"1. With respect to the causes of varieties, or the circumstances under which they originate, or in connexion with which they may be found more or less uniformly to exist, we are not yet prepared for drawing any conclusions. It will be the object of the succeeding parts of this work to investigate the physical history of all the races of men which form the population of different countries. In the course of this investigation, it will appear how far their physical characters are found in connexion with particular influences of climate and situation; whether or not they have local relations, and how far they may be connected with peculiarities of habit and the manner of life. At the conclusion of this enquiry, the proper place will be found for entering, with a prospect of success, on the consideration above suggested.

"2. The enquiry which regards the permanency of varieties is the most difficult investigation which the physical history of mankind presents; and those physiologists who have maintained the original diversity of races, have rested their argument entirely on this ground. This subject also will, as I trust, be elucidated in the succeeding parts of my work, in which I shall endeavour to trace by ethnographical researches how far the peculiar traits of different races are constant, and in what degree they have deviated from the original or prevalent character of each tribe; and to discover examples, if any such exist, in which important alterations of figure and complexion have originated within the limits of history. In adverting briefly to the comparison of different species in this particular point of view, we may observe, that varieties in the tribes of animals are very different in regard to the constancy of their transmission. In some species they are much more regularly handed down to the offspring than in others: this is a well-known fact in relation to the vegetable tribes, and it has been already noticed and illustrated by Mr. Knight and other writers on the propagation of plants. In animals the same difference is observable: in certain cases varieties are continually springing up, and they as speedily disappear. Perhaps the sheep may serve for one instance of these more variable tribes, in which likewise the varieties which arise are more likely to become again lost. In certain species, varieties are, as it would appear, produced with greater difficulty; and in these, when once generated, they continue with greater permanency. In some instances it is not improbable that modifications in the form and texture of parts, originating but rarely, become in future time constant and invariable characters. We have, however, in all instances sufficient evidence that the same law of hereditary transmission prevails in every tribe, by which qualities belonging to individuals, such at least as are congenital, have a tendency to preserve themselves in propagation, and to become appropriated to whole progenies. This is the principle on which the existence of permanent varieties depends; and we have already found sufficient evidence of its agency, both in human races and in many of the tribes of animals and plants." (P. 373.)

Our anxious desire not to misstate an argument pursued through a volume has induced us to quote this passage at length; and the reader will gather from it the nature of the contents of the volume, yet unpublished, which is to succeed it.

In connexion with the negative evidence of the unity of the human species, drawn from the fact that no remarkable instance of variation is found among mankind, to which a parallel may not be found in the lower orders of the creation, Dr. Prichard reminds us, in conclusion, of the evidence deduced in the course of his enquiry in the earlier parts of his volume: these have been already noticed by us, and, deferring further notice of the subject until the appearance of the second volume, we think it is scarcely necessary for us to assure the medical student that, for a philosophical view of the whole question of man's origin and varieties, as far as human knowledge can throw light upon either, and for various and interesting information, industrious enquiry, careful reasoning, and just conclusions, he will find no work superior, and few works equal to that which has occupied our attention in this article. Dr. Prichard's fame has long been spread far beyond the limits of his profession and nation, and the well-earned honours he has gained both abroad and at home reflect back lustre upon both.

---

